# Protein–Protein Interaction (PPI) Network of Zebrafish Oestrogen Receptors: A Bioinformatics Workflow

**DOI:** 10.3390/life12050650

**Published:** 2022-04-27

**Authors:** Rabiatul-Adawiah Zainal-Abidin, Nor Afiqah-Aleng, Muhammad-Redha Abdullah-Zawawi, Sarahani Harun, Zeti-Azura Mohamed-Hussein

**Affiliations:** 1 Malaysian Agricultural Research & Development Institute (MARDI), Serdang 43400, Malaysia; rabiatul@mardi.gov.my; 2 Institute of Marine Biotechnology, Universiti Malaysia Terengganu, Kuala Nerus 21030, Malaysia; 3 UKM Medical Molecular Biology Institute (UMBI), Universiti Kebangsaan Malaysia, Kuala Lumpur 56000, Malaysia; mraz@ukm.edu.my; 4 Centre for Bioinformatics Research, Institute of Systems Biology (INBIOSIS), Universiti Kebangsaan Malaysia, Bangi 43600, Malaysia; sarahani@ukm.edu.my; 5 Department of Applied Physics, Faculty of Science and Technology, Universiti Kebangsaan Malaysia, Bangi 43600, Malaysia

**Keywords:** bioinformatics, oestrogen receptor, network analysis, protein–protein interaction, network, zebrafish

## Abstract

Protein–protein interaction (PPI) is involved in every biological process that occurs within an organism. The understanding of PPI is essential for deciphering the cellular behaviours in a particular organism. The experimental data from PPI methods have been used in constructing the PPI network. PPI network has been widely applied in biomedical research to understand the pathobiology of human diseases. It has also been used to understand the plant physiology that relates to crop improvement. However, the application of the PPI network in aquaculture is limited as compared to humans and plants. This review aims to demonstrate the workflow and step-by-step instructions for constructing a PPI network using bioinformatics tools and PPI databases that can help to predict potential interaction between proteins. We used zebrafish proteins, the oestrogen receptors (ERs) to build and analyse the PPI network. Thus, serving as a guide for future steps in exploring potential mechanisms on the organismal physiology of interest that ultimately benefit aquaculture research.

## 1. Introduction

The advancement of omics technologies, such as genomics, transcriptomics, proteomics, and metabolomics, has produced high throughput datasets to identify molecules associated with the physiological mechanisms of interest. However, identifying associated molecules without knowing their interactions is inadequate to comprehend the mechanisms underlying the presented physiology [1]. In addition, cellular physiology is rarely governed by a single protein but rather by a group of interacting proteins. This subcellular interaction has been driven by protein–protein interaction (PPI) to understand better the mechanisms underlying the given physiology [2].

Investigating the PPIs can provide better insights into the molecular machinery in a cell. PPIs have various roles, including modulating the kinetic characteristics of enzymes, catalysing metabolic events, activating or repressing proteins, altering the specificity of proteins, regulating upstream and downstream levels, and transporting molecules [3,4]. Given the critical importance of PPIs in organismal physiology, targeting PPIs involved in specific biological processes and responsible for phenotypic variation is an effective technique, especially in assisting molecular breeding and disease pathogenesis in aquaculture [5].

### 1.1. Protein–Protein Interaction (PPI)

PPI is a study of how proteins work together in a cell to perform cellular functions in a coordinated manner [6]. PPIs can be measured using two different experimental techniques, such as in vitro and in vivo. High-throughput techniques, such as tandem affinity purification-mass spectroscopy (TAP-MS), affinity chromatography and protein array, are examples of in vitro techniques in PPI detection [7]. Affinity purification methods are based on the specificity of antibody–epitope interaction [8]. The yeast-2-hybrid (Y2H) [9,10] and synthetic lethality are in vivo techniques. According to Fields and Song [11], the Y2H is scalable and can be used to evaluate the interaction of several proteins in parallel with some automation.

PPI network is an organisation of interacting proteins produced by biochemical events that serve a specific biological function as a complex [12]. A comprehensive PPI network has been developed using experimental resources, such as the Y2H and TAP-MS. However, due to the labour-intensive and time-consuming PPI detection via an experimental method, the computational analysis of the PPI network is becoming more popular for predicting PPIs from various characteristics of proteins. In the PPI network, proteins are described as nodes, and their relationships (i.e., physical or functional interactions) are described as edges. It is widely known that the edge direction of the PPI network is usually undirected, and the edge weight is usually unweighted [13]. However, weighted edge evidence in the PPI network can be valued either using experimental or computational approaches.

In general, the PPI network has been used in various biological analyses: (i) to assign putative roles of uncharacterised proteins, (ii) to characterise the relationships between proteins that form multi-molecular complexes, and (iii) to identify the biological pathways that are related to similar proteins [14]. PPI has been utilised in biomedical research to unveil the complex pathogenesis of human diseases. Human diseases, such as cancers, polycystic ovarian syndrome (PCOS), cardiovascular diseases, and diabetes, are governed by more than one protein and are involved in several biological processes and pathways [15,16,17,18,19]. Studying the related proteins with their partners facilitates: (i) identifying genes or proteins responsible for the diseases in a network-based approach, (ii) determining subnetworks related to particular biological processes, and (iii) searching for new genes or proteins related to the diseases. The PPI network has also been applied in plants to predict the function of unknown proteins [6,20,21], deduce putative mechanisms that relate to signal transduction, homeostasis control, stress responses, plant defence, development, and organ formation that are contributed to crop improvement [22,23]. The ‘guilt-by-association principle’ has been used in the PPI network to infer the function of unknown or poorly characterised proteins in a cluster of protein networks [24]. Hence, the PPI network can be integrated with the functional annotation workflow and shows the importance of integrative analysis in understanding biological mechanisms.

Similar efforts can be performed in aquaculture research as the PPI network can contribute knowledge related to molecular aspects to this field. For instance, the PPI network adopted in human diseases can also be used to understand the diseases that plague the aquaculture industry by identifying the proteins responsible for aquatic diseases (i.e., white spot disease in the penaeid shrimps that cause by a white spot syndrome virus (WSSV)) [25,26]. It can also be used to predict the pathogenesis of the diseases, which is essential to improve disease prognosis and diagnosis and design targeted antibacterial drugs in Nile tilapia, *Oreochromis niloticus* [27]. In addition, the functional annotation of unknown proteins in aquaculture species can also be predicted using the PPI network [25]. However, PPI information on aquaculture species remains limited except for a model organism of zebrafish, *Danio rerio.*

Zebrafish have become an essential organism since 1960. Classically, it has been used as a translational model to study human genetics and diseases due to high genomics and molecular similarities with humans (i.e., at least 75% similarity to human genes) [28,29]. In the past two decades, zebrafish has also been used as a model with great utility in various aquaculture studies, including growth and reproduction [30,31], nutrition [32], and diseases and immune responses [33]. Due to its relevance in broad research topics, various data, including PPI, on zebrafish are available and publicly accessible for extensive studies on both biomedical and aquaculture. For instance, more than 10,000 protein-coding genes have been annotated in zebrafish, which will enable the prediction of poorly-characterised protein in aquaculture species using PPI network analysis [34]. A study has constructed a PPI network between *Candida albicans* and zebrafish to understand the disease pathogenesis mechanism towards facilitating the development of new antifungal drugs [35]. In another study, the PPI network of the fifth chromosome of zebrafish was constructed as a model to understand the growth and developments in the model organism [36]. Hence, the wealth of zebrafish PPI information has provided new insights into improving the fish aquaculture industry. In this review, the PPI network on one of the sex steroid hormones, oestrogen receptors, will be used to exemplify the integration of several resources in finding the interacting partners of the proteins of interest. Oestrogen receptors are among the most studied nuclear receptors in zebrafish and play important roles in aquaculture species, especially vertebrates, as they mediate the activity of endocrine-disrupting chemicals that can cause imbalanced endogenous hormones to the exposed organisms by regulating hormone synthesis and metabolism [37]. The relevant knowledge obtained from the PPI network will be highlighted in this review.

### 1.2. Protein–Protein Interaction Databases

The number of known PPIs has increased significantly in recent years. The accumulation of PPI data supports the construction of PPI networks and allows systematic and holistic studies based on the PPI network. Several publicly accessible databases have been established to gather and store PPI data to make this knowledge more accessible. To date, several PPI databases have been developed to provide PPI data, such as Biological General Repository for Interaction Datasets (BioGRID) [38], Database of Interacting Proteins (DIP) [39], GeneMANIA [40], IntACT [41], Molecular Interaction Database (MINT) [42], and the Search Tool for Retrieval of Interacting Genes/Proteins (STRING) [43]. These PPI databases provide an integrated web interface for searching and exploring the experimental and computational PPIs.

GeneMANIA and STRING store both experimental and computationally predicted PPI information (i.e., co-expression, co-occurrence, protein homology, gene neighbourhood and gene fusion) [40,43]. DIP, BioGRID, and MINT compile PPI data from publications that identify PPI using experimental methods [39,42]. PPI databases, such as IntAct and IMEx, integrate PPI data from the publications and other sources from PPI databases [41,44]. A recently developed database, the Integrated Interactions Database (IID) [45], focuses on the tissue-specific PPIs that would facilitate the experimental studies in model organisms. Table 1 summarises the PPI databases containing PPI information in zebrafish.

**Figure 1:**
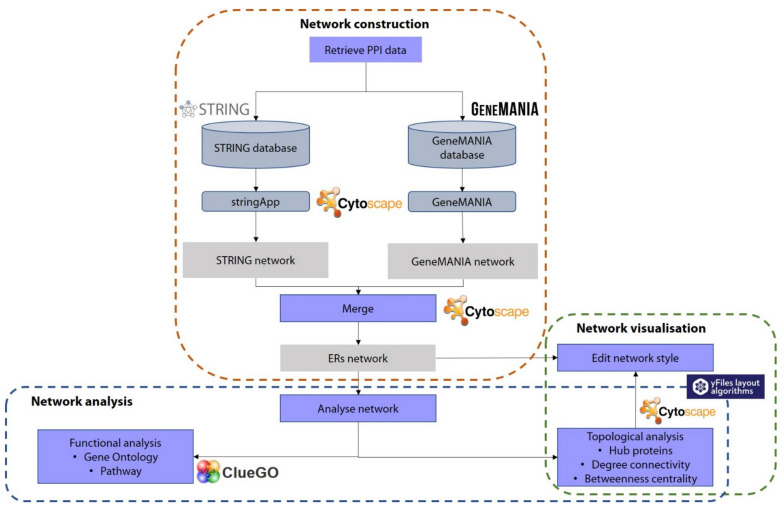
Bioinformatics workflow for the construction of protein–protein interaction network (PPI). Each step is included in the dotted square. The purple box represents the step, the blue shape denotes the database or tool, and the grey box represents the generated result.

The main idea of this review is to start constructing the PPI network by retrieving the relevant PPI data using public databases and performing general analysis of the constructed PPI network. Figure 1 summarises the workflow for constructing a PPI network using several bioinformatics tools and PPI databases discussed in this review. The bioinformatics workflow for the PPI network is structured in three steps. The first step is to construct the network by retrieving and merging the PPI data from public databases embedded in the Cytoscape. The second step involves the improvement of the network visualisation by editing the style of the network. Finally, the third step is to analyse the network using topological and functional analyses. This review only describes the functional analysis in detail as this analysis is able to extract meaningful biological information from such a PPI network. However, this review briefly provides how to retrieve the topological results and shows how the results can be used to improve the network visualisation.

## 2. Bioinformatics Workflow for Protein–Protein Interaction Network

### 2.1. Network Construction and Visualisation Platform Using Cytoscape

Cytoscape version 3.8.2 was used as the network integration, analysis, and visualisation platform [46]. Cytoscape is a state-of-the-art and open-source software that can be run on Windows, Mac, and Linux platforms with the requirement of Java installation. It can be freely downloaded via the Cytoscape website (https://cytoscape.org/download.html (accessed on 15 July 2021)). A wide range of Cytoscape apps is available for different types of analysis, such as network clustering (i.e., MCODE [47], ClusterViz [48]), network enrichment (i.e., ClueGO [49], BiNGO [50], ENViz [51], ReactomeFIViz [52]), and pathway analysis (i.e., KEGGScape [53], WikiPathways [54]). These Cytoscape apps can be installed through Application Manager, which can be found in the Apps tab of the Cytoscape header. The Cytoscape app can also be installed and extensively familiarised from the App Store website (https://apps.cytoscape.org/ (accessed on 15 July 2021)). Cytoscape is also embedded in *NetworkAnalyzer*, a tool that can calculate the topology, network density, and connectivity of nodes and edges [55].

Several tools also have been developed to construct and visualise the PPI network, such as Gephi [56], MEDUSA [57], Arena 3D [58], Protein Interaction Network Visualizer (PINV) [59]. Gephi is an open-source platform for network visualisation and can handle many datasets, of which up to 100,000 nodes and 1,000,000 edges. Gephi is a standalone network visualisation. It facilitates network analysis, such as calculating clustering coefficients, shortest paths, and node degree. MEDUSA is developed based on the Java application. MEDUSA also provides clustering algorithms (i.e., k-Means, spectral) for module detections in a PPI network. Arena 3D visualises and links the networks that contain different types of biological information in a three-dimensional space. PINV is a web-based PPI network visualisation, which does not require an installation process. It provides several PPI datasets, i.e., host–pathogen, disease, and drug, that can be visualised using this web-based tool. Although each network visualisation tool has distinctive features in terms of graphical representation, the ultimate goal is to join or link the proteins together, forming a PPI network. Table 2 summarises the abovementioned tools used to perform PPI network analysis in zebrafish.

### 2.2. Retrieving PPIs of Oestrogen Receptors (ERs) from Public PPI Databases

The PPI data in this review were retrieved from STRING and GeneMANIA, as both databases contain a large number of PPI datasets, including experimental and predicted interactions. Integrative analysis by combining data from different databases is essential to obtain a comprehensive PPI network and a complete biological system model [60]. The more data from various sources that are integrated, the more informative the PPI network is. The interaction information in the PPI databases is assigned with the interaction score representing the confidence value of interaction. Three oestrogen receptors (ERs) have been found in zebrafish, namely ERalpha, ERbeta2, and ERbeta1, encoded by *esr1*, *esr2a* and *esr2b*, respectively [61]. These ERs are required to mediate the activities of oestrogen, which is a sex steroid hormone that plays a role in various physiological processes in both reproductive and nonreproductive tissues of zebrafish [62]. In teleosts, ESR1/*esr1* (ERalpha) has vertebrates homologs, ESR2a/*esr2a* (ERbeta2) is conserved with mammalian, and ESR2b/*esr2b* (ERbeta1) shows no homology across mammalian, resulting in the unclear function of ESR2b in zebrafish [63]. Hence, investigating the interactions of the ERs in zebrafish might reveal a better understanding of ERs functions in zebrafish and other teleosts.

**Figure 2:**
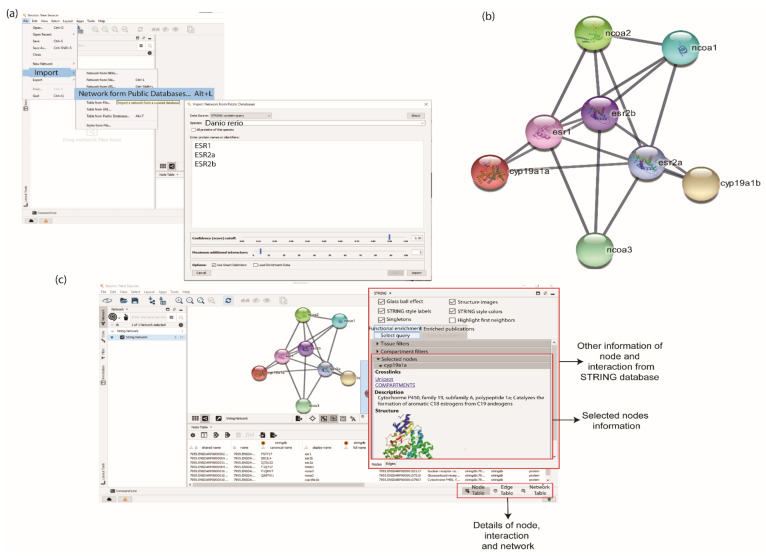
Retrieving the oestrogen receptors (ERs) protein and their interaction partner from STRING database. (**a**) User can insert the protein names or identifiers, select the confidence score and maximum interactors. By providing this information, STRING will search the interaction network among proteins of interest. (**b**) The interaction network of proteins using the STRING database. (**c**) Nodes and edges information are provided at the bottom table. Detailed information from the STRING database is shown in the right panel.

To retrieve interaction partners of ERs using STRING and GeneMANIA database, the apps of stringApp and GeneMANIA must be initially installed from the Application Manager of Cytoscape by clicking ‘Apps > App Manager’. Both apps can be searched in the Search box of the App Manager window. The Install button can be clicked once a specific app is selected (Figure S1). Users can click ‘File > Import > Network from Public Databases’. A pop-up box will appear, and the user can choose ‘Data Source and Species’ (Figure 2a). To retrieve PPI from the STRING database, the user can choose ‘STRING: protein query’ in the dropdown list. In this study, *D. rerio* was selected as ‘Data Source and Species’ in the STRING pop up box. The ERs protein names or identifiers, namely ESR1, ESR2a, and ESR2b, were inserted in the protein names and identifier box. The confidence score was set at a high confidence value, 0.9, to remove the false positive interaction. The maximum additional interactors, which determines the number of interaction partners of the ERs, was set to 5. After all the parameters were selected, the PPI network of the ERs was generated by clicking the Import button (Figure 2a).

A total of eight proteins or nodes, including ESR1, ESR2a, and ESR2b, with 20 interactions/edges were constructed using STRING. The STRING network listed proteins that interact with all inserted protein queries (Figure 2b). All details on node and edge produced in the Cytoscape panels were displayed at the bottom table. Users can retrieve further information on the nodes by clicking on a ‘Specific node’ dropdown option located at the right panel of the Cytoscape window (Figure 2c).

**Figure 3:**
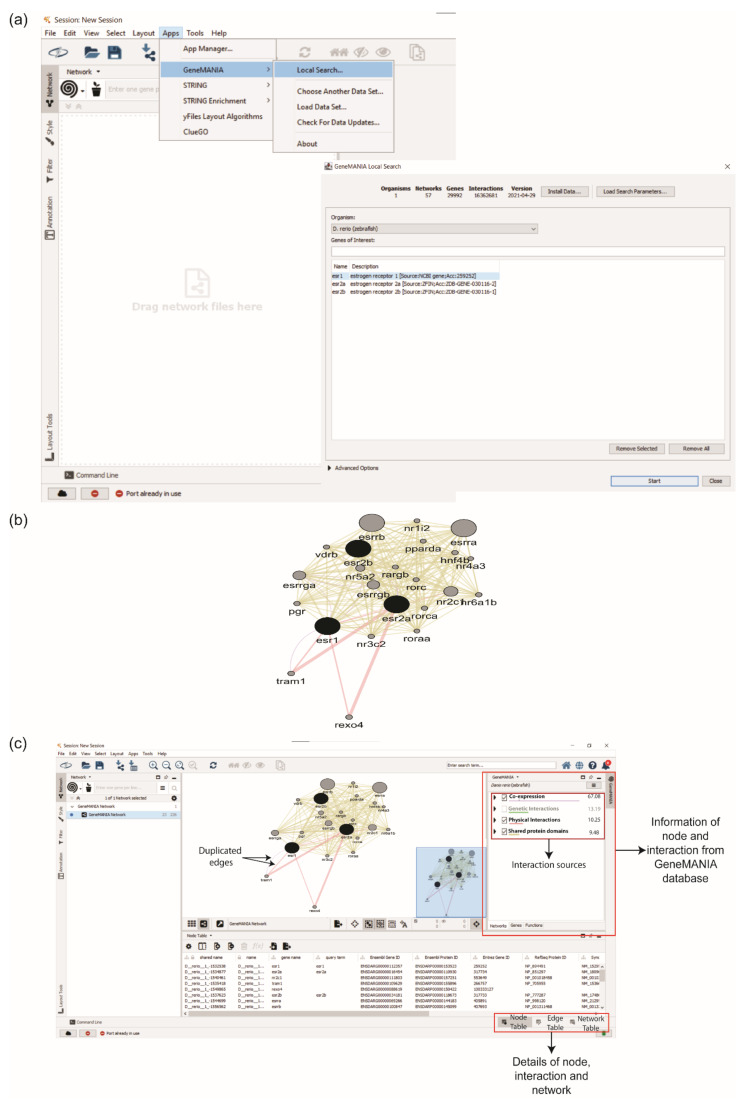
Retrieving protein–protein interaction (PPI) network using GeneMANIA. (**a**) User insert gene or protein of interest in the ‘Gene of Interest’ box. (**b**) PPI network of ESR1, ESR2a, and ESR2b. (**c**) Nodes and edges information are displayed in the right interface. Examples of duplicated edges were labelled on the interaction between esr1 and tram1, where the colour of each edge represents the interaction sources, i.e., Co-expression (purple) and Physical Interactions (red).

ESR1, ESR2a, and ESR2b were inserted in the ‘Gene of Interest’ box to obtain the interaction partners of ERs protein from GeneMANIA (Figure 3a). The number of interactors can be set from the ‘Advanced Options’. The generated PPI network consisted of 23 proteins with 226 interactions (Figure 3b). This PPI network contains duplicated edges or interactions, which refers to the interactions that link similar protein partners. Duplicated edges exist in the GeneMANIA network because each edge represents a different source of interactions, such as physical interactions, co-expressions, etc. (Figure 3c).

The second approach to retrieve the interaction partners of protein is using the ‘Search’ function in the PPI web server. For example, STRING (http://STRING.org (accessed on 1 July 2021)) allows the users to retrieve the interaction partners by typing the ESR1, ESR2a and ESR2b in the ‘Search’ box. The organism of interest can be selected from the ‘Species’ drop-down list, in which zebrafish will be selected in this case. GeneMANIA (http://genemania.org (accessed on 1 July 2021)) also allows the users to search for the interaction partners of the individual or multiple proteins (recommended for less than 100 proteins) in a particular organism, which can be found at the ‘Search’ box at the top right of the homepage. The generated network data from STRING and GeneMANIA can be downloaded and imported in .tsv and .txt format, respectively. Both formats can be exported to .xls format and then imported into Cytoscape. The details of the second approach are displayed in Figures S2 and S3. The advantage of using GeneMANIA and STRING via the Cytoscape app enables the users to retrieve a large number of proteins.

### 2.3. PPI Networks Integration

A Cytoscape app, ‘Merge’, was used to merge PPI networks generated by STRING and GeneMANIA into an integrated network. User can click ‘Tools’ > ‘Merge’ > ‘Network’. Before merging, a column of the database was added into the node table for each network, and the column of data type was into the edge table only for the STRING network. This step is necessary to distinguish which databases identified which proteins and edges. The STRING and GeneMANIA networks from the ‘Available Networks’ were moved into ‘Networks to Merge’ and accomplished the integration of the network.

**Figure 4:**
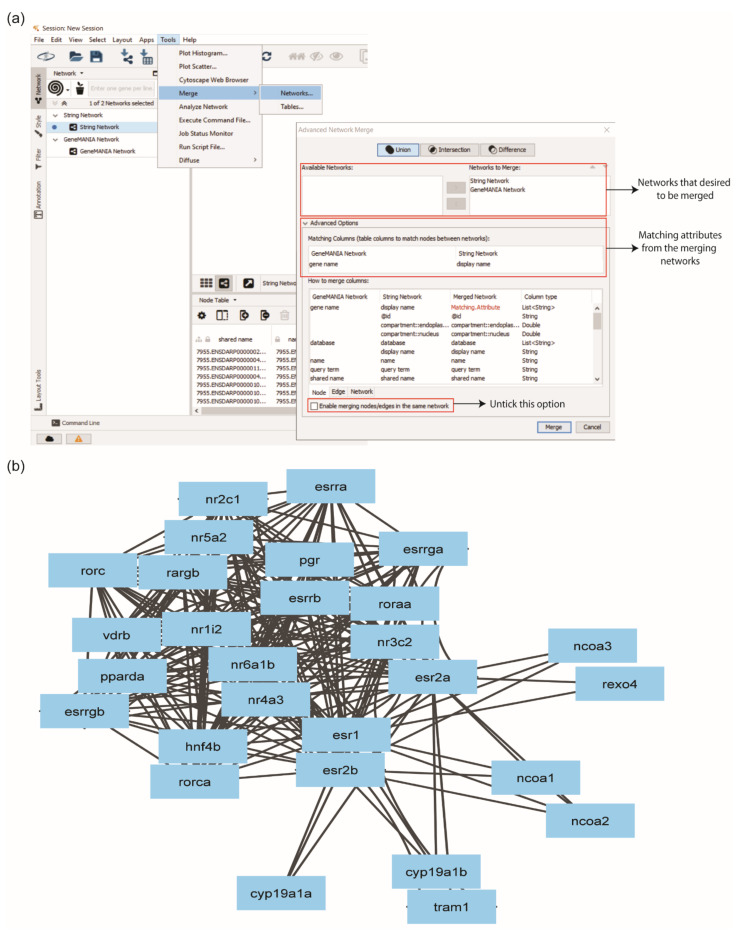
Merging multiple sub-networks using the ‘Merge’ option in Cytoscape. (**a**) User must select a similar identifier among the sub-networks to enable the merge process. (**b**) Protein–protein interaction network of ERs with 28 nodes and 234 edges.

Each protein in a network has a shared name, which is the key identifier. Since the proteins shared names from both networks differed, similar node attributes from the node table were determined as matching attributes. The determination of matching attributes in the merged network was performed at the ‘Advanced Options’. Display name of STRING network and gene name of GeneMANIA were selected as matching attributes. The ‘Enable merging nodes/edges in the same network’ box was unticked to retain the duplicated edges from the GeneMANIA network (Figure 4a). Otherwise, the information of edges will be automatically eliminated. The merged network was renamed ERs network, generated 28 proteins with 234 interactions (Figure 4b). The ERs network showed that different interaction partners were identified from STRING and GeneMANIA databases. Hence, the integration of PPI from different databases is essential to obtain the comprehensive interaction information of the protein of interest.

In network integration, data integration errors could occur due to attribute data files that are not properly integrated with the networks. The possible cause is that the gene identifier columns in the two networks do not match perfectly. Hence, the user must double-check that the node table has similar gene identifiers to integrate the PPI networks.

### 2.4. Editing PPI Network Style

**Figure 5:**
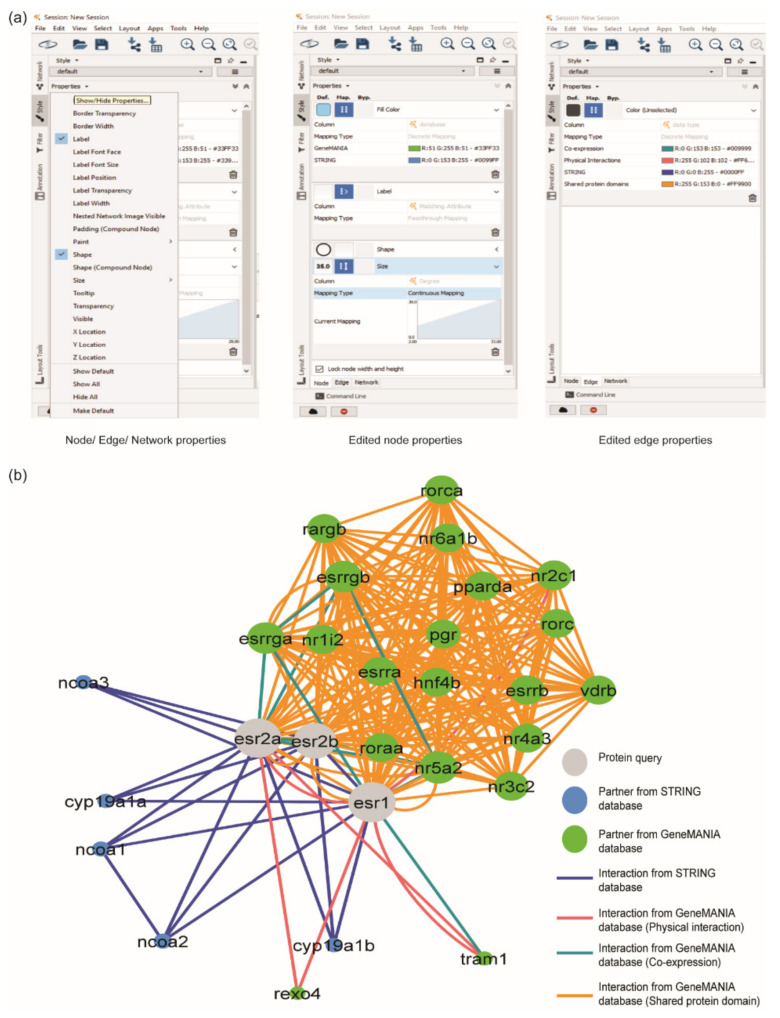
Editing the style of PPI network. (**a**) Node, edge, and network properties can be edited by exploring the ‘Style’ option. (**b**) PPI interaction network of ERs protein, after editing the nodes and edges properties. The grey circle represents ERs protein, the blue circle represents the protein interactor from STRING database, and the green circle represents the protein interactor from GeneMANIA database.

This method is critical for visualising the network and communicating essential information of the generated network. Each property (node, edge, and network) of the network can be edited at the ‘Style’ option, located on the left side of Cytoscape (Figure 5a). All nodes were set to ‘circle’ shape by clicking the circle at the default option, the first box inside the shape option. The label of the nodes was changed to ‘Matching attribute’. The colour of nodes was set to ‘discrete mapping’ based on the column database, which is a column that was added in the PPI networks integration, by clicking the second box inside the fill colour option. The interaction partners from STRING were assigned as blue, and GeneMANIA was green. Protein queries (i.e., ESR1, ESR2a, and ESR2b) were assigned with grey by manually selecting those proteins and selected the grey colour from the bypass option, the third box inside the fill colour. A similar step was performed for the edge colour. Any edge properties can be edited by clicking the edge button at the bottom of the ‘Style’ viewer. The colour of the edges can be adjusted by checking the ‘Edge colour to arrows’ using discrete mapping based on the data type column.

The size of the node was set based on the number of interactions in the network. To determine the number of the interaction of each node, users can click ‘Tools’ > ‘Analyse Network’, then click ‘Uncheck Analyse as Directed Graph’. The number of interactions was displayed at the column Degree of the node table. The node’s size was assigned by checking ‘lock node width and height’ and selecting the continuous mapping style based on the ‘Degree’ column.

The proteins in the network were automatically organised by selecting ‘Layout’ > ‘yFiles Organic Layout’. This layout can be adopted by installing the app of yFiles Layout Algorithms by clicking ‘Apps > App Manager’. yFiles Layout Algorithms provides eight types of layouts, where each layout portrays different meanings (Figure S4). In this review, Organic Layout was selected because this layout algorithm is a multi-purpose layout style for the undirected network. Figure 5b shows the final results of the merged network.

### 2.5. Functional Analysis

The functional analysis involved the functional annotation and enrichment of the proteins in the network. Gene ontologies (GO) terms (i.e., biological process, molecular function, and cellular component) and pathway are the most common enrichment analyses. The functional analysis plays a role in interpreting the network into biological function. In this analysis, the Cytoscape app, namely ClueGO coupled with CluePedia, were used. ClueGO requires a license that can be freely requested at the ClueGO website (http://www.ici.upmc.fr/cluego/cluegoLicense.shtml (accessed on 20 July 2021)) [49]. In ClueGO, the functional categories of zebrafish were downloaded, and each category was updated to obtain the latest datasets.

**Figure 6:**
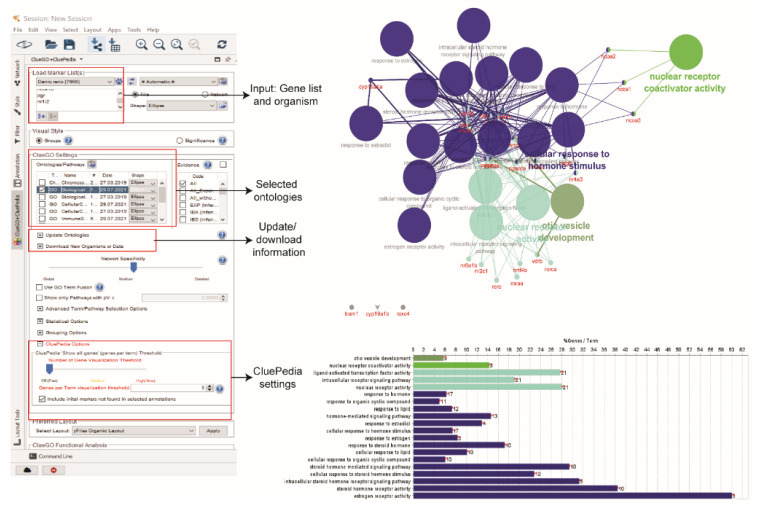
Biological process enrichment analysis using ClueGO and CluePedia.

The gene names from the node table of ERs network were pasted into the ‘Load Marker List(s)’ box. For the biological process (BP) enrichment analysis, default ClueGO settings were used. At the CluePedia Options, the box of ‘Include initial markers‘ that were not found in selected annotations was checked. At the CluePedia panel of the ClueGO and CluePedia table, the option of ‘Show genes’ that form initial clusters was selected to visualise the proteins that link to enriched BP (Figure 6).

**Figure 7:**
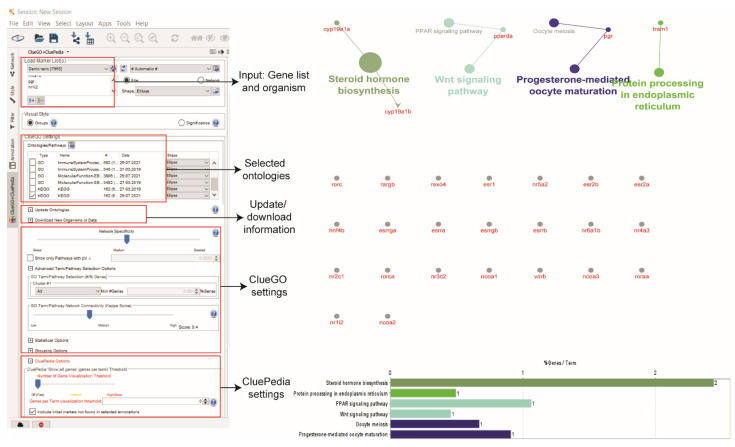
KEGG pathway enrichment analysis using ClueGO and CluePedia.

The ‘Advanced Term/Pathway’ selection option in ClueGO can be changed from ‘3’ to ‘All’. This selection will result in the list of any pathways (including insignificant) related to the proteins in the ERs network (Figure 7). The stringApp also provides functional annotation and enrichment analysis. This analysis can be performed at the Cytoscape results panel and ‘Apps’ > ‘STRING Enrichment’. Besides that, GeneMANIA also provides GO annotations on each protein in the network.

In functional enrichment analysis, one gene may be associated with several GO terms and pathways. The statistical tests are used to calculate over-representation analysis of GO terms and pathways, such as Fisher’s exact test, hypergeometric distribution, and followed by multiple testing (i.e., *p*-value correction), including Bonferroni and Benjamini-Hochberg, to reduce the false-positive rate of the significant GO terms and pathways [64]. GO terms and pathways with a corrected *p*-value less than the cut-off of 0.05 will be considered significant biological properties.

## 3. Discussion and Future Direction

The PPI network is a valuable method to organise, integrate, and analyse large-omics scale data sets generated from the omics platform (i.e., transcriptomics, proteomics and metabolomics). Generally, omics data provide a list of molecules (i.e., genes, proteins, and metabolites) that might be involved in specific physiology. They ignore the interaction information between the listed molecules. The interaction information is valuable for predicting the potential mechanisms of the aetiology and physiology of interests [5]. Hence, this review will assist the researchers who are interested in exploring their datasets using the PPI network approach.

In this study, the ERs network shows that each PPI database (i.e., STRING, GeneMANIA) covers different PPI network data. Integrating the interaction data from several PPI databases is essential to obtain high coverage of the ERs partners. Nevertheless, it is vital to filter the interaction with a high confidence score as provided by the STRING database. However, the interaction among the protein does not necessarily infer them to physically bind with one another because most of the interaction criteria (i.e., co-occurrence, co-expression and textmining) only predict the interaction among proteins. A high confidence score might reduce the false positive interactions by removing the interactions that might not interact in an actual situation. The experiments, such as pull-down assays [65], co-immunoprecipitation (co-IP) [66], far-Western blot analysis [67] and crosslinking [68], are among examples that can be adopted to validate the in silico interactions.

Functional analyses are important to interpret the biological meanings of the PPI network. In this review, GO enrichment analysis identifies 20 significant biological processes that enriched the ERs network. Biological process enrichment analysis shows most of the ERs interaction partners are involved in similar biological processes. The ERs network can be further analysed, for example, by integrating the network with a knowledge-based approach to construct the putative mechanisms of the processes involved in oestrogen regulation in zebrafish, such as embryonic development [69,70], sex differentiation [71], and reproductive processes [72]. In addition, the significant BPs from the enrichment analysis may further support the function of ERs in silico interaction-based evidence partners participating in the important processes in the zebrafish.

The pathway enrichment analysis shows no significant pathways enriched the ERs network, probably due to the limited pathway information of the proteins in the ERs network that was extracted from the Kyoto Encyclopedia of Genes and Genomes (KEGG) database [73]. Reducing the ClueGO parameters might give clues on the ERs functions in zebrafish. According to the guilt-by-association principle, the involvement of the interaction partners of ERs in the pathways of the Wnt signalling pathway, oocyte meiosis, steroid hormone biosynthesis, peroxisome proliferator-activated receptor (PPAR) signalling pathway, progesterone-mediated oocyte maturation and protein processing in the endoplasmic reticulum suggests the potential involvement of ESR1, ESR2a, and ESR2b in these pathways, and possible association of these pathways in the process that relate to oestrogen regulation in zebrafish [74,75]. ERs played a significant role in regulating early Wnt signalling in the presence or absence of ESR1 [76] and exhibit cell-dependent transcription activities during oocyte meiosis in female reproductive organs [77]. Limited information of ERs in these enriched pathways may shed light on their promising function that could become a target for future aquaculture research.

Other than functional analysis, topological analysis is one of the approaches often used to analyse the network. For instance, the interaction between nodes can be analysed to explore descriptive network properties such as degree distribution (number of edges connected to a node), neighbourhood connectivity (connectivity of neighbours), clustering coefficient (how nodes are connected in their neighbourhood), and betweenness centrality (how much this node controls other nodes) [78]. *NetworkAnalyzer* in the Cytoscape has been widely used to calculate the network metrics. It computes many centrality metrics to assist in identifying important nodes in a network [55].

A degree is the number of connections (edges) a node has to other nodes. Nodes with a high degree are called hubs, and these hubs tend to exert a large amount of control on the network compared with a node with fewer connections [79]. A highly connected protein node may indicate a master regulator of a specific biological process [80]. Neighbourhood connectivity is the average connectivity of all neighbours of a given node. Betweenness centrality calculates how central a node is within a network and indicates the node’s level of influence on its neighbours and the network as a whole [78]. The connections between protein nodes provide functional information about the relationship between those genes or proteins. It is widely accepted that those interacting genes are more likely to share a similar function or be involved in a similar biological pathway or process, a principle known as guilt-by-association [80]. Although several datasets did not show any correlations between network topology and biological meanings [74], many recent studies applied this approach to analyse the constructed network. This approach manages to improve understanding by highlighting the involvement of several proteins in a specific function, which are beneficial to enhancing the medical and agriculture sectors [81,82,83,84].

This tutorial review reveals a PPI network construction and analysis workflow using available software. However, there is no unique method, and each network may require specific software, especially in analysing the complex PPI data. Integration of PPI data from various databases highlights the similarities and differences in the PPI datasets. Hence, the challenge in this integrative analysis is to recognise the similar identifier in each network and choose the correct parameters, which will lead to identifying the best network and candidate genes and proteins for further study.

Integrative network analysis using multi-omics data continues to evolve. Thus, the associated bioinformatics tools related to PPI network construction, analysis, and enrichment need to be updated accordingly. To date, the analysis tools (i.e., network construction, visualisation, and analysis) proposed in this review have been provided with a convenient and user-friendly interface. More PPI data from aquaculture species are also needed to be deposited in public databases, improving the current PPI databases into a data-rich database platform. These efforts will enhance the PPI network approach, which can improve the understanding of complex systems biology in aquaculture, such as host–pathogen interaction.

## 4. Conclusions

This review exemplifies the construction of a PPI network using multiple existing PPI databases that contain the molecular interaction data of zebrafish. The ERs of zebrafish were used as protein queries to provide a molecular interaction required in facilitating the activities of oestrogen in zebrafish. The integration of interaction information generated from different PPI databases (GeneMANIA and STRING) successfully captures extensive interaction partners of the ERs. The Cytoscape app has been utilised to improve the visualisation and the analysis of the generated PPI network. Functional analysis unfolds the biological meanings of the network. Investigating the PPI of ERs or other proteins allows researchers to better understand their roles in the context of a biological system, which may then be applied to molecular-assisted breeding to improve aquaculture practices. Although the data we used in this review were retrieved from public databases, the workflow here should be applicable to work with protein data from any aquaculture species. We expect this review will reach and assist beginner-level scientists in exploring PPI networks without the need for programming skills, while also encouraging them to enhance the field further.

## Figures and Tables

**Table 1:** Summary of protein–protein interaction (PPI) databases that contain PPI information in zebrafish.

Database	Description	URL (Reference)
Biological General Repository for Interaction Datasets (BioGRID)	Provides molecular interaction data from a comprehensive curation approach by experts. It contains PPI information for most model organisms, exceeding 70 species in total.	https://thebiogrid.org/ (accessed on 12 August 2021) [38]
Database of Interacting Proteins (DIP)	Stores experimentally verified PPIs identified by curators from published articles.	https://dip.doe-mbi.ucla.edu/dip/Main.cgi (accessed on 12 August 2021) [39]
GeneMANIA	Facilitates functional inference using genomics (GEO) and proteomics (BioGRID, IRefIndex, and I2D) molecular data. It currently houses nine model organisms (*Arabidopsis thaliana, Caenorhabditis elegans, Danio rerio, Drosophila melanogaster, Escherichia coli, Homo sapiens, Mus musculus, Rattus norvegicus* and *Saccharomyces cerevisiae*).	https://genemania.org/ (accessed on 1 July 2021) [40]
IntAct	Provides analysis for molecular interaction data. All interactions are derived from literature curation and user submissions.	https://www.ebi.ac.uk/intact/ (accessed on 12 August 2021) [41]
Molecular Interaction Database (MINT)	Contains experimentally verified PPIs extracted from literature curation mined by experts. The interaction data of 667 species can be generated from this database.	https://mint.bio.uniroma2.it/ (accessed on 12 August 2021) [42]
STRING	A powerful database that integrates known and functional predicted associations between molecular data. The upcoming STRING version 11.5 will provide more than 14,000 organisms in the repository.	https://string-db.org/ (accessed on 1 July 2021) [43]
IMEx	A database that serves curated and non-redundant protein interaction acquired from several databases of published peer-reviewed journals, such as MINT, IntAct, and DIP.	https://www.imexconsortium.org/ (accessed on 22 May 2021) [44]
Integrated Interactions Database (IID)	A database that provides resources on tissue-specific PPIs in a human and non-model organism (i.e., mouse, fly, rat, worm). This database integrates known, experimental, and predicted PPIs.	http://iid.ophid.utoronto.ca/ (accessed on 12 August 2021) [45]

**Table 2:** Summary of selected tools that can be used to construct, analyse, and visualise the PPI network information in zebrafish.

PPI Tools	Type of Application	Description	URL (Reference)
Cytoscape	Standalone	A powerful tool that enables visualisation, interpretation, and integration of myriads biological interaction networks derived from heterogeneous data. It also provides a wide range of network analysis apps for the data import from public databases, enrichment, graph analysis, topological, gene ontology, and clustering.	https://cytoscape.org (accessed on 15 July 2021) [46]
MCODE	Cytoscape app	An automated app that detects the highly connected regions in large protein interaction networks. The molecular complexes are indicated as clusters/subnetworks/groups/modules and always depict important insights into many biological conditions.	https://apps.cytoscape.org/apps/mcode (accessed on 22 May 2021) [47]
ClusterViz	Cytoscape app	Searches molecular complexes in a PPI network using three distinct clustering algorithms of FAG-EC, EAGLE, and MCODE.	https://apps.cytoscape.org/apps/clusterviz (accessed on 22 May 2021) [48]
ClueGO	Cytoscape app	Detects enriched functional modules in a network. The functional module can be Gene Ontology and pathway.	https://apps.cytoscape.org/apps/cluego (accessed on 20 July 2021) [49]
BiNGO	Cytoscape app	Investigates significant Gene Ontology in a set of genes of the PPI network.	https://apps.cytoscape.org/apps/bingo (accessed on 22 May 2021) [50]
ENViz	Cytoscape app	Performs Gene Ontology and pathway enrichment analysis on expression datasets of miRNA, non-coding RNA, and proteins.	https://apps.cytoscape.org/apps/enviz (accessed on 22 May 2021) [51]
ReactomeFIViz	Cytoscape app	Interestingly, also known as Reactome Cytoscape Plugin or ReactomeFIPlugIn. It helps to investigate the relationship between proteins using enrichment analysis, referring to Reactome pathways.	https://apps.cytoscape.org/apps/reactomefiplugin (accessed on 22 May 2021) [52]
KEGGScape	Cytoscape app	Enables users to manually recreate the pathway diagrams using reference pathways retrieved from the KEGG database. It also incorporates annotations and experimental data into pathways that help clarify the biological systems.	https://apps.cytoscape.org/apps/keggscape (accessed on 22 May 2021) [53]
WikiPathways	Cytoscape app	Allows users to import biological pathways from the WikiPathways database, integrate with experimental omics data, and visualise them in Cytoscape.	https://apps.cytoscape.org/apps/wikipathways (accessed on 22 May 2021) [54]
NetworkAnalyzer	Cytoscape app	Interprets the PPI network through the topological analysis, including node degrees, shortest paths, clustering coefficient, and neighbourhood connectivity.	https://apps.cytoscape.org/apps/networkanalyzer (accessed on 22 May 2021) [55]
Gephi	Standalone	An open-source tool for visualising and interpreting molecular interaction networks. It also provides topological functions such as network centrality measures and density, average path, and clustering coefficient.	https://gephi.org/ (accesed on 22 May 2021) [56]
MEDUSA	Java standalone	Analyses heterogeneous data from multiple sources into a single network and includes a variety of clustering methods for more insightful interpretation and visualisation.	https://sites.google.com/site/medusa3visualization/ (accessed on 22 May 2021) [57]
Arena 3D	Webtool	Composes multilayered graphs in 3D to visualise interactions between numerous types of data and groups of the highly interconnected region.	http://bib.fleming.gr:3838/Arena3D/ (accessed on 22 May 2021) [58]
Protein Interaction Network Visualizer (PINV)	Webtool	An interactive tool for visualising PPI networks and provides a function to manipulate the colour of the protein nodes based on their cellular functions.	http://biosual.cbio.uct.ac.za/pinv.html (accessed on 22 May 2021) [59]

## Data Availability

Not applicable.

## Supplementary Materials

The following supporting information can be downloaded at: https://www.mdpi.com/article/10.3390/life12050650/s1, Figure S1: Installation of ‘stringApp and ‘GeneMania’ apps; Figure S2: An alternative approach to constructing a PPI network from the STRING website; Figure S3: An alternative approach to constructing a PPI network from the GeneMANIA website; Figure S4: ERs network with different layouts generated by the app of yFiles Layout Algorithm.
