# Supplementary material for: Protein–Protein Interaction (PPI) Network of Zebrafish Oestrogen Receptors: A Bioinformatics Workflow
Source: Life (Basel). 2022 Apr 27;12(5):650. doi: 10.3390/life12050650 (PMC9143887; doi:10.3390/life12050650)
Supplement: Supplementary file 1 [file life-12-00650-s001.zip › Figure S1.pdf]

Figure S1:

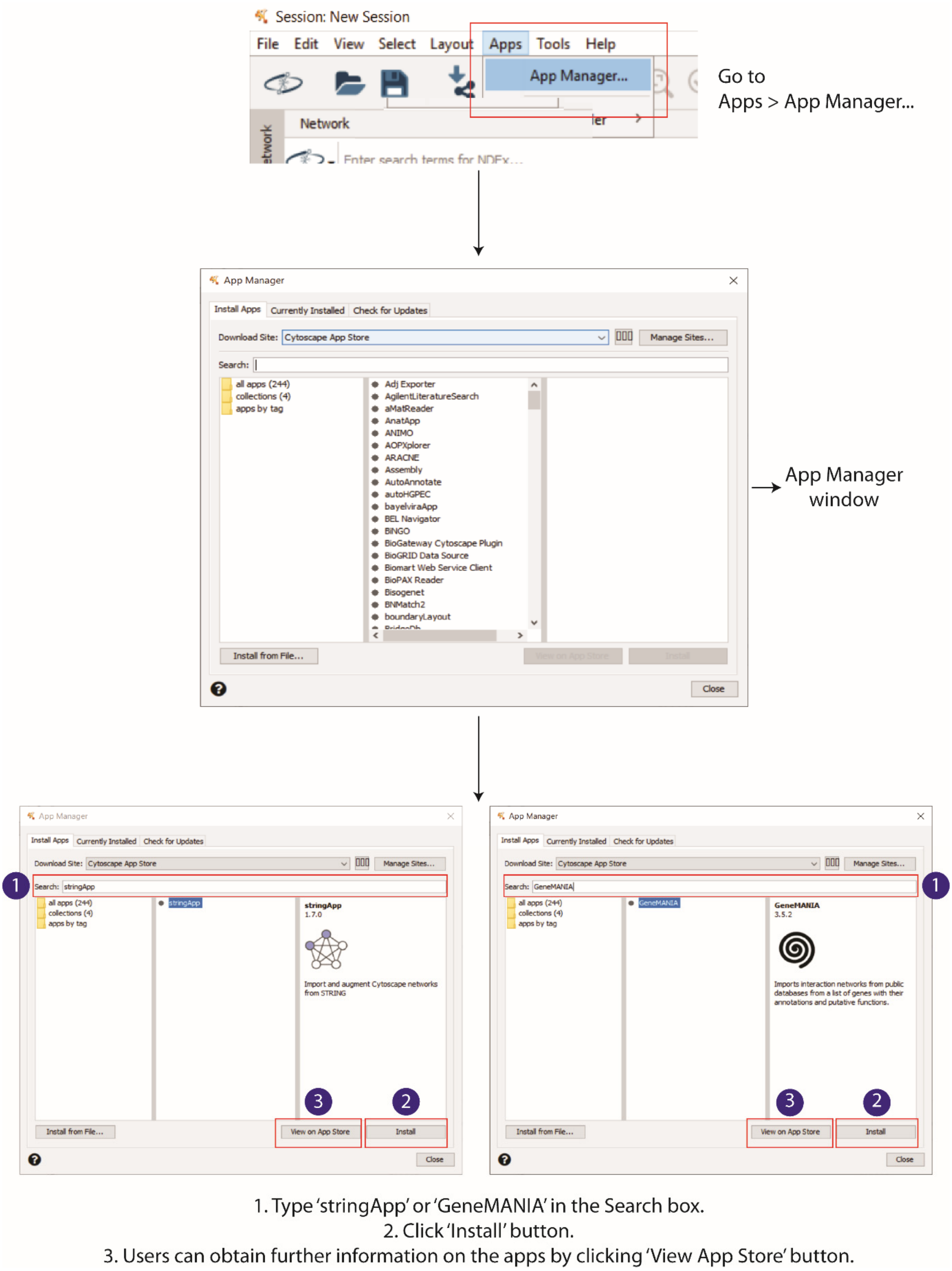

Figure S1: Installation of 'stringApp and 'GeneMania' apps. Users can apply similar steps to install other Cytoscape apps.
