# Supplementary material for: Protein–Protein Interaction (PPI) Network of Zebrafish Oestrogen Receptors: A Bioinformatics Workflow
Source: Life (Basel). 2022 Apr 27;12(5):650. doi: 10.3390/life12050650 (PMC9143887; doi:10.3390/life12050650)
Supplement: Supplementary file 1 [file life-12-00650-s001.zip › Figure S2.pdf]

Figure S2:

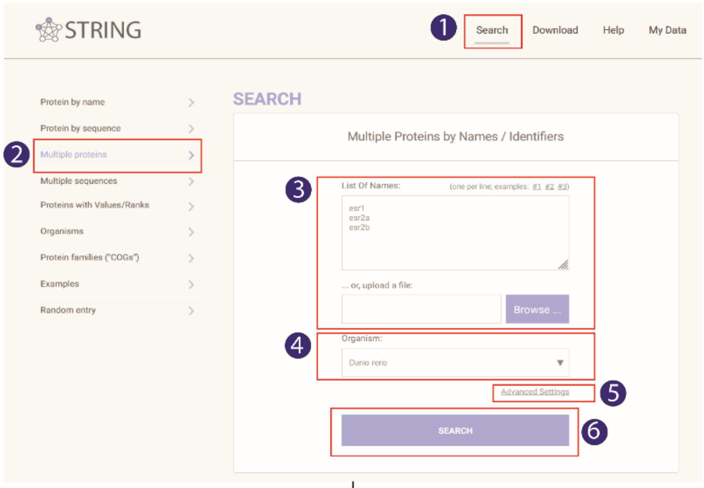

1. On STRING database website, users can click 'Search' menu.
2. Select 'Multiple proteins'.
3. Insert the input in the 'List of Names' box. Users can alsoupload the file that contains the input using 'Browse' button.
4. Select the specific organism.
5. Users can click on 'Advanced Settings' to edit the network settings.
6. Click 'Search' button to proceed.

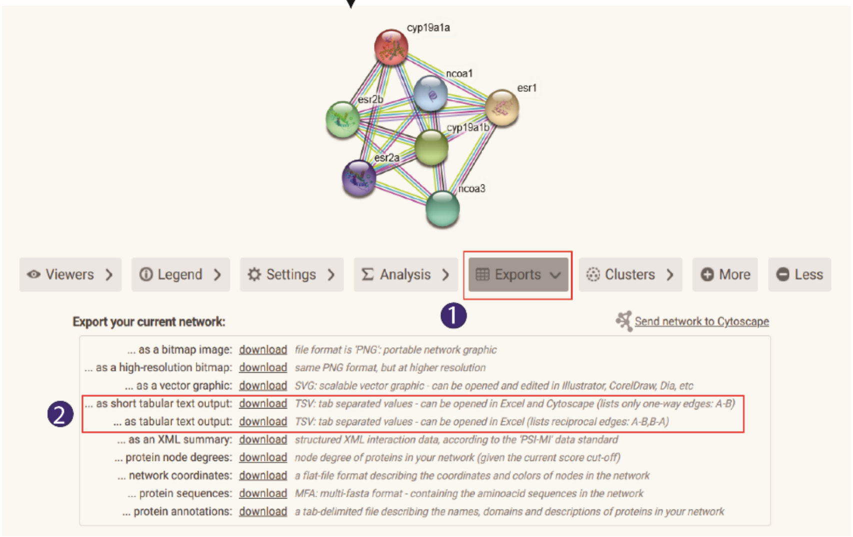

1. Click 'Export' button.
2. Select .tsv format to download the interaction information.

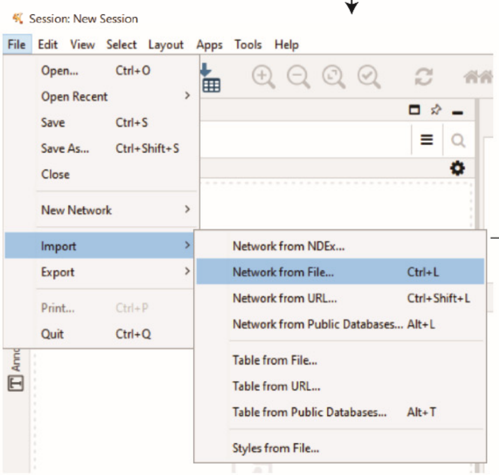

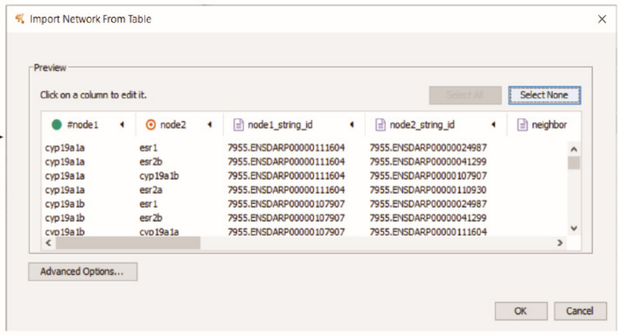

Go to Cytoscape, click 'File > Import > Network from File. Select the .tsv file that have been downloaded.

This window will appear. User can click on a column to edit the column information. Click 'OK' to generate a PPI network.

Figure S2: An alternative approach to constructing a PPI network from the STRING website.
