# Supplementary material for: Protein–Protein Interaction (PPI) Network of Zebrafish Oestrogen Receptors: A Bioinformatics Workflow
Source: Life (Basel). 2022 Apr 27;12(5):650. doi: 10.3390/life12050650 (PMC9143887; doi:10.3390/life12050650)
Supplement: Supplementary file 1 [file life-12-00650-s001.zip › Figure S3.pdf]

Figure S3:

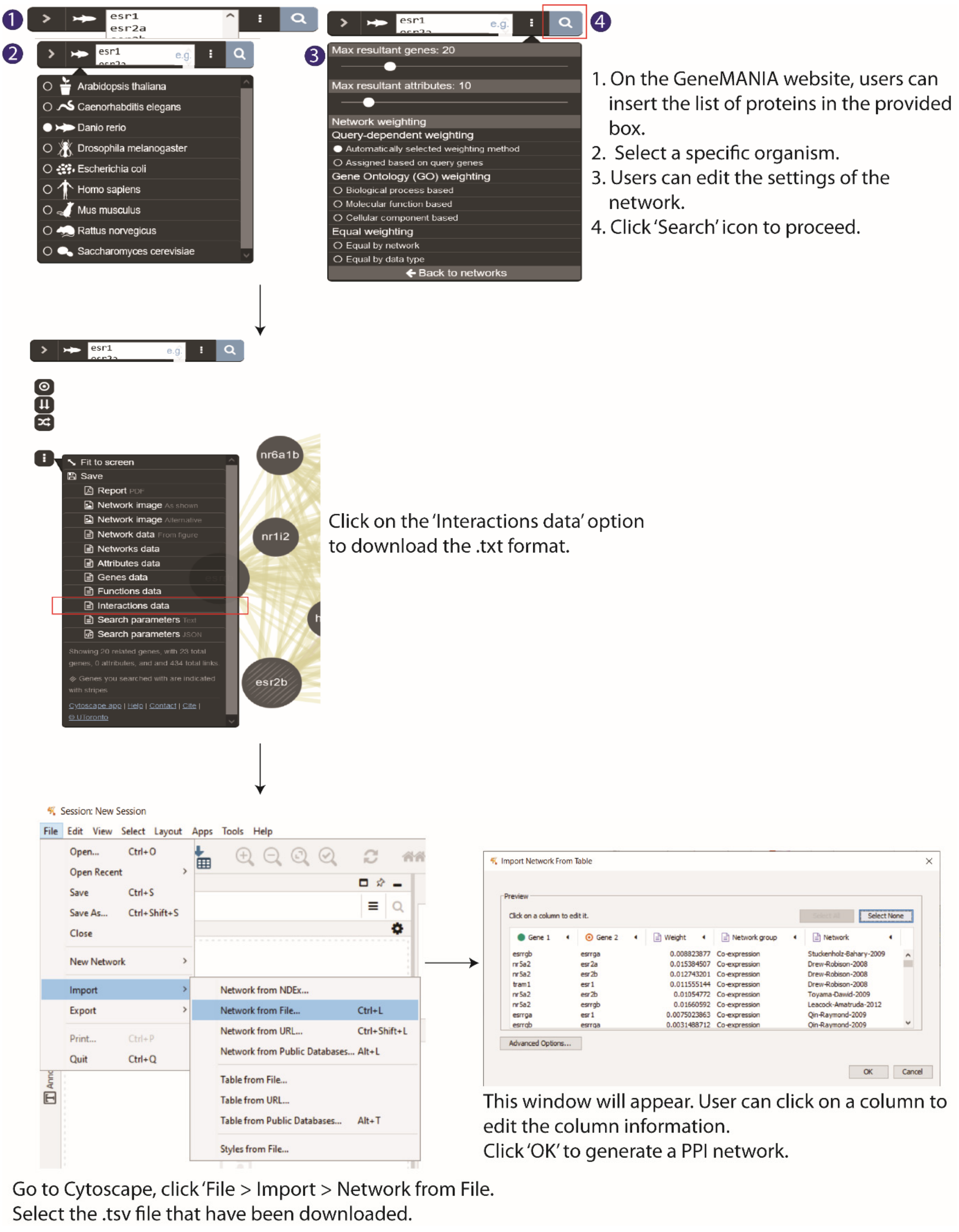

Figure S3: An alternative approach to constructing a PPI network from the GeneMANIA website.
